# Specific plasmid patterns and high rates of bacterial co‐occurrence within the coral holobiont

**DOI:** 10.1002/ece3.3717

**Published:** 2018-01-11

**Authors:** Deborah C. A. Leite, Joana F. Salles, Emiliano N. Calderon, Jan D. van Elsas, Raquel S. Peixoto

**Affiliations:** ^1^ Institute of Microbiology Federal University of Rio de Janeiro Rio de Janeiro Brazil; ^2^ Genomics Research in Ecology and Evolution in Nature ‐ Groningen Institute for Evolutionary Life Sciences University of Groningen Groningen The Netherlands; ^3^ NUPEM/Macaé Federal University of Rio de Janeiro Rio de Janeiro Brazil; ^4^ Instituto Coral Vivo Santa Cruz Cabrália Brazil; ^5^ IMAM‐AquaRio – Rio Marine Aquarium Research Center Rio de Janeiro Brazil

**Keywords:** co‐occurrence, corals, holobiont, mobile genetic elements, plasmids

## Abstract

Despite the importance of coral microbiomes for holobiont persistence, the interactions among these are not well understood. In particular, knowledge of the co‐occurrence and taxonomic importance of specific members of the microbial core, as well as patterns of specific mobile genetic elements (MGEs), is lacking. We used seawater and mucus samples collected from *Mussismilia hispida* colonies on two reefs located in Bahia, Brazil, to disentangle their associated bacterial communities, intertaxa correlations, and plasmid patterns. Proxies for two broad‐host‐range (BHR) plasmid groups, IncP‐1β and PromA, were screened. Both groups were significantly (up to 252 and 100%, respectively) more abundant in coral mucus than in seawater. Notably, the PromA plasmid group was detected only in coral mucus samples. The core bacteriome of *M. hispida* mucus was composed primarily of members of the Proteobacteria, followed by those of Firmicutes. Significant host specificity and co‐occurrences among different groups of the dominant phyla (e.g., Bacillaceae and Pseudoalteromonadaceae and the genera *Pseudomonas*,* Bacillus,* and *Vibrio*) were detected. These relationships were observed for both the most abundant phyla and the bacteriome core, in which most of the operational taxonomic units showed intertaxa correlations. The observed evidence of host‐specific bacteriome and co‐occurrence (and potential symbioses or niche space co‐dominance) among the most dominant members indicates a taxonomic selection of members of the stable bacterial community. In parallel, host‐specific plasmid patterns could also be, independently, related to the assembly of members of the coral microbiome.

## 1 INTRODUCTION

Corals can harbor complex microbial ecosystems, which frequently result in the development of both specific and variable host‐associated microbial communities (reviewed in Webster & Reusch, 2017), which can benefit host fitness (Peixoto, Rosado, Leite, Rosado, & Bourne, 2017; Webster & Reusch, 2017). Despite the close relationship between corals and their associated microbiomes, which can include organisms that have effects that vary from beneficial (Damjanovic, Blackall, Webster, & van Oppen, 2017; Krediet, Ritchie, Paul, & Teplitski, 2013; Peixoto et al., 2017; Webster & Reusch, 2017) to pathogenic (Meistertzheim, Nugues, Quéré, & Galand, 2017; Sweet & Bulling, 2017; Wright et al., 2017), knowledge of these intrinsic symbiotic, or dysbiotic, that is, disrupted symbiotic relationships (Bosch & Miller, 2016; Egan & Gardiner, 2016; Petersen & Round, 2014), interactions, and associated mechanisms is sparse.

It has been proposed that important mechanisms associated with the holobiont, that is, the host and its associated microbial community (Margulis & Fester, 1991), can be regulated through microbiome shuffling (i.e., shifts in microbial abundance) and/or switching (i.e., acquisition of the microbial strains from the surrounding environment) (reviewed in Webster & Reusch, 2017). The acquired microorganisms could also be passed on from parental to offspring generations (Leite et al., 2017; Padilla‐Gamiño, Pochon, Bird, Concepcion, & Gates, 2012). This microbiome‐mediated transgenerational acclimatization (MMTA) (proposed by Webster & Reusch, 2017) could lead to the rapid adaptation (and evolution) of corals to adverse environmental conditions. This natural acclimatization could be boosted in the face of environmental stresses (Damjanovic et al., 2017; Peixoto et al., 2017), for example, through the manipulation of specific key members of the microbiome, which have recently been termed “beneficial microorganisms for corals” (BMCs) (Peixoto et al., 2017). However, several questions remain, namely who are these key beneficial players, is there a taxonomic selection of the dominant microbes, and how do they interact within the holobiont?

Knowledge of the patterns of variation and interactions within the coral microbiome is limited. Other microbial‐community studies have shown that evaluation of co‐occurrence patterns in microbiomes may offer a more comprehensive view of complex microbial communities, constituting a complementary approach to estimates of alpha and beta diversity (Barberán, Bates, Casamayor, & Fierer, 2012; Dini‐Andreote et al., 2014). Identifying microbial patterns (Andrade et al., 2012; Peixoto et al., 2011; Rachid et al., 2013; Santos, Cury, Carmo, Rosado, & Peixoto, 2010) and potential interactions among microorganisms may reveal stable populations and shared niches, indicating preferences for certain resources, and consequently, microbial groups that are more competitive for such niches, or even elucidating potential direct symbiotic relationships between these microorganisms (as suggested by Barberán et al., 2012). This approach may be especially promising in coral microbiome studies because the close relationship between the host and its microbial community reported in several studies (Ainsworth, Thurber, & Gates, 2010; Cárdenas, Rodriguez‐R, Pizarro, Cadavid, & Arévalo‐Ferro, 2012; Ceh, Keulen, & Bourne, 2013; Ceh, Raina, Soo, van Keulen, & Bourne, 2012; Kelly et al., 2014; Lema, Bourne, & Willis, 2014; Lins‐De‐barros et al., 2010, 2013; Mouchka, Hewson, & Harvell, 2010; Sharp, Ritchie, Schupp, Ritson‐Williams, & Paul, 2010; Thompson, Rivera, Closek, & Medina, 2014). We believe, in particular, that exploring the taxonomic diversity of the bacterial part of the microbiome core (the bacteriome) as well as relevant ecological rules shaping these communities could provide valuable tools to guide BMC and MMTA surveys.

Another potential key aspect of coral microbiomes that has not received much attention is horizontal gene transfer (HGT) and the presence of specific patterns to support gene exchange. HGT plays important roles in bacterial evolution and gene exchange (Bhattacharya et al., 2016; van Elsas, Turner, & Bailey, 2003; Heuer & Smalla, 2007). Conjugation, for instance, which is mediated by different classes of mobile genetic elements (MGEs), allows the acquisition of novel genes (Heuer & Smalla, 2012). Plasmids, which are the main vectors for this genetic exchange, can act in the acquisition of genes or genetic pathways (such as for antibiotic resistance, pollutant degradation, and others) (Dealtry et al., 2014; Heuer & Smalla, 2012; Izmalkova et al., 2006). This HGT could be advantageous for holobiont resilience under environmental disturbance and, therefore, constitute a key component for MMTA (Webster & Reusch, 2017). Despite their possible essential role, plasmid patterns are largely unexplored in corals.

In this study, we present a survey of proxies for two broad‐host‐range (BHR) plasmid groups, IncP‐1B and PromA, in *Mussismilia hispida* coral mucus and the surrounding seawater. These plasmids can efficiently transfer their genetic material to a wide range of hosts and have been widely used as proxies to evaluate the potential spread of genes in several environments (van der Auwera et al., 2009; Heuer & Smalla, 2007, 2012; Zhang, Pereira e Silva, Chaib De Mares, & Van Elsas, 2014) and as providers of bacterial HGT capacities in some soil environments (Zhang et al., 2014). We also describe the bacterial diversity in these samples, as well as the co‐occurrence patterns of the coral bacteriome. We discuss the potential impact of these results in the context of the MMTA.

## 2 MATERIAL AND METHODS

### 2.1 Ethics approval and consent to participate

Permission for sampling was obtained from the Brazilian Institute of the Environment and Renewable Natural Resources (IBAMA)/Chico Mendes Institute for Biodiversity Conservation (ICMBio), permanent permit number 16942, in accordance with the Normative Instruction No. 03/2014 of System Authorization and Information on Biodiversity (SISBIO), and from local authorities of the Municipality Environmental Agency (SMMA), Porto Seguro, Bahia, Brazil. The microbial survey permit was obtained from CNPq (National Council for Scientific and Technological Development).

### 2.2 Sampling procedures and total DNA extraction

Mucus samples (around 50 ml) were collected with syringes directly from the polyps of *M. hispida* colonies on two reefs located adjacent to a marine protected area (Parque Natural Municipal do Recife de Fora) of Porto Seguro, Bahia, Brazil, in January 2015, as described by Castro et al. (2010). Particular microhabitats, for instance the surface mucus layer (SML), function as physical and chemical barriers (Shnit‐Orland & Kushmaro, 2009) that corals can benefit from, using antimicrobial compounds and their endogenous microbiome to regulate bacterial colonization (Ritchie, 2006), as the SML closely interacts with the surrounding environment. For instance, Lee, Davy, Tang, Fan, and Kench (2015) observed shifts in the relative abundance of the genera *Endozoicomonas* and *Vibrio* during a bleaching event and suggested that these changes resulted in a decrease in coral health, as a consequence of the increased ability of potentially pathogenic bacteria to pass through the SML barrier. Based on these observations, the SML is likely a potential source of MGEs and a favorable microhabitat for HTE.

Samples were obtained at the following sites: (1) Recife Itassepocu, 2 km from the mouth of the Buranhém River (6°25.9′46.37″S, 039°01′19.42″W) (closer to the river), totaling four samples from morphologically healthy colonies (without white spots) and four samples from colonies with morphological alterations (with white spots) and (2) Recife de Fora, 9.4 km from the Buranhém River mouth (16°23′23.72″ S, 038°58′54.92″W) (more distant from the river), totaling four mucus samples from morphologically healthy colonies. Sampling was performed in quadruplicate, so that each colony constituted a replicate. Approximately 1,000 ml of surrounding seawater (10–50 cm from the colony) (four replicates at each site) was also collected and filtered through a 0.22‐μm filter, using a standard vacuum pump system (Prismatec 131B). All samples (mucus and filters) were immediately immersed in liquid nitrogen and then stored at −80°C in the laboratory. Mucus samples and seawater material scraped off the filters were homogenized, and the DNA was extracted using the PowerSoil^®^ DNA Isolation Kit (MoBio Laboratories, Carlsbad, CA, USA) following a modification of the method described by Sunagawa, Woodley, and Medina (2010).

### 2.3 Bacterial diversity

The V4 variable region of the 16S rRNA gene from all samples was amplified using the primers 515F/806R (Caporaso et al., 2011), and paired‐end (2 × 250 bp) sequencing was performed at the Argonne National Laboratory in their Next Generation Sequencing Core, on an Illumina Miseq, following the manufacturer's guidelines. The QIIME software package (version 1.9.1) was used to process the raw sequence data (Caporaso et al., 2010b). In brief, sequences were trimmed using the following parameters: quality score >25, sequence length >200, maximum homopolymer length of 6, and 0 mismatched bases in the primers and barcodes.

The remaining high‐quality sequences were binned into operational taxonomic units (OTUs) at 97% sequence identity using USEARCH 6.1 (v6.1.544) followed by selection of a representative sequence for each OTU (Edgar, 2010). Chimeric sequences were also identified using USEARCH 6.1 (v6.1.544) (Edgar, 2010) and removed. A representative sequence for each phylotype was aligned against the Greengenes database (Desantis et al., 2006), using PyNAST (Caporaso et al., 2010a), with sequences classified through the Greengenes taxonomy using the RDP classifier (Wang, Garrity, Tiedje, & Cole, 2007). Before further analysis, singletons, chloroplast plastids, mitochondria, and archaeal sequences were removed from the dataset. For all OTU‐based analyses, the original OTU table was rarified to a depth of 22,900 sequences per sample to minimize the effects of sampling effort on the analysis. The QIIME package was also used to generate weighted UniFrac distance matrices (Lozupone, Hamady, & Knight, 2006) and α‐diversity metrics, including richness and diversity indices. All sequences were deposited in the NCBI Sequence Read Archive database, with the accession numbers SRR5903387–SRR5903406.

In this study, we considered “core” as the set of bacterial taxa universally present in all samples, as defined by Shade and Handelsman (2012) and Turnbaugh et al. (2007). Considering that these microbes are common across microbiomes, they could be capable of playing key roles in a given ecosystem (Shade & Handelsman, 2012; Turnbaugh et al., 2007). The mucus‐core bacteriome was identified using QIIME and determined by plotting the OTU abundance, and it was represented by OTUs shared by 100% of the samples.

Network analyses were conducted on a subset of coral mucus microbiomes (sites 1 and 2) from *M. hispida,* using both the 179 most abundant OTUs (obtained after filtering rare taxa, i.e., sequences <0.005%) and the core bacteriome OTUs. Significant correlations between OTUs with a minimum occurrence of 10 were determined using the following metrics: Pearson's correlation, Spearman's correlation, and Bray–Curtis dissimilarity, using the CoNet app (Faust & Raes, 2012) in Cytoscape v.3.0.2 (Shannon et al., 2003). Networks obtained by all analyses were merged by intersection, keeping only interactions that were supported by all methods. The measurements were performed with 1000 iterations. The Benjamini–Hochberg multiple test correction was applied, and clusters (highly interconnected regions) were identified using the MCODE application (Bader & Hogue, 2003).

### 2.4 Quantification of bacterial, plasmid and integron genes

Quantitative PCR was used to estimate the gene copy numbers per ml of 16S rRNA genes; class I integrons, which were used as a proxy for anthropogenic pollution (Gillings et al., 2015) and BHR, and IncP‐1β and PromA plasmid groups, from mucus and seawater samples (Table S1). DNA preparations from plasmids R571 and pTer331 were used as positive controls in the detection of IncP‐1β and PromA group plasmids, respectively, and DNA from plasmid R388 was used as a positive control to detect plasmids of the IncW group and class I integrons. DNA from the IncQ plasmid RSF1010 was used as a negative control for all PCRs. Quantitative PCR experiments were conducted in an ABIPrism 7300 (Applied Biosystems) detection system, following the manufacturer's recommendations. Amplification of all genes was performed in a 20 μl reaction volume, containing 10 μl of GoTaq^®^ q‐PCR Master Mix 2× (Promega), primers, 0.02 μl T4 gene 32 protein (5 mg/ml), H_2_O, and 2–5 ng DNA. The temperature profile included an initial hot start for 3 min at 94°C; and PCR cycling and detection (40 cycles) for 1 min at 94°C, 45 s at the stated annealing temperature (Table S1), and 45 s at 72°C (acquiring signal at the end of this step). All samples were used in triplicate, and H_2_O was used as the negative control. The primers and qPCR conditions used are summarized in Table S1. The efficiency and melting curves from all reactions were determined and analyzed using the ABIPrism 7300 Detection System (Applied Biosystems). For all genes, the cut‐off value was <10^2^.

### 2.5 Statistical analyses

Estimates of α‐diversity and β‐diversity were based on an evenly rarified OTU abundance matrix. Statistical differences of qPCR analyses and α‐diversity matrices (observed OTUs, phylogenetic distance—PD, and the Chao index) were determined using analysis of variance (ANOVA) followed by a Tukey post hoc test.

To analyze the difference between the profiles and compositions of the bacterial communities, we used a principal coordinates analysis—PCoA (Jolliffe, 1986), using a Bray–Curtis distance matrix with PRIMER6 (Kelly et al., 2015). To assess the variation among different samples (coral mucus and seawater), we used a permutational multivariate analysis of variance (PerMANOVA) (Kelly et al., 2015) using PRIMER6 and PERMANOVA+ (Anderson, Gorley, & Clarke, 2008). Similarity percentage (SIMPER) calculations were conducted using PRIMER6 (Kelly et al., 2015) based on Bray–Curtis dissimilarity, in order to define the OTUs primarily responsible for the differences among the groups.

## 3 RESULTS

### 3.1 Bacterial community structure

**Figure 1:**
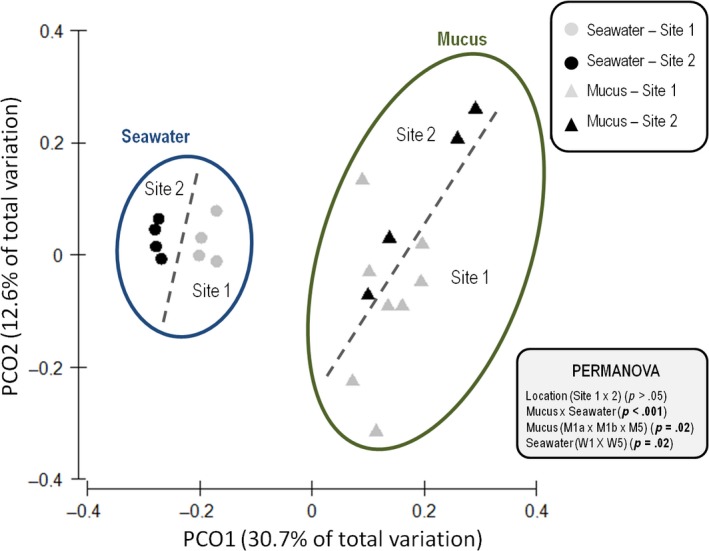
NMS (Bray–Curtis) plot of bacterial communities associated with *Mussismilia hispida* mucus and surrounding seawater at Recife Itassepocu (Site 1) and Recife de Fora (Site 2) based on 16S rRNA gene sequence data (*n *=* *4). Contours and dashed lines are based on significant pairwise PERMANOVA results (*p *=* *.01). Circle represents seawater replicates, and triangle represents mucus replicates. Light gray indicates samples from site 1, and black indicates samples from site 5

The bacterial communities detected in the coral mucus differed from those in the seawater, as evidenced using PCoA unweighted UniFrac analyses of the data (Figure 1). The replicates of each of the two biomes clustered together, whereas the biomes themselves were clearly separate. Pairwise PERMANOVA of the data confirmed that the microbial communities of the mucus and seawater were significantly different from each other (*p *<* *.001), but detected no significant effect of location (sampling sites 1 × 2, all samples together). However, the location had some influence on the microbial communities from mucus (*p *=* *.024) and seawater (*p *=* *.027). No significant differences (*p* > .05) were observed between the microbiome structures from healthy colonies (without white spots) and colonies with morphological alterations (with white spots), from all sites. The richness values also differed, with the highest bacterial richness observed in the seawater samples from site 2 (Table S2).

Regarding the identity of the OTUs, a suite of diverse bacterial taxa was observed. Proteobacteria was the most abundant phylum associated with the coral mucus and seawater microbiomes, followed by Bacteroidetes and Firmicutes (Figure S1), and the core bacteriome was basically represented by Proteobacteria (Figure S2). The 10 most abundant OTUs represented around 58%–67% of the entire mucus bacteriome at site 1, and 38% at site 2. The top 10 OTUs consisted of members of the genera *Pseudoalteromonas*,* Halomonas,* and *Rugeria*, and of the families Pseudoalteromonadaceae, Rhodobacteraraceae, Desulfovibrionaceae, Desulfobulbaceae, Hyphomonadaceae, Acidaminobacteriaceae, and Flavobacteriaceae (Figure S3).

The top 23 (83.55%) OTUs responsible for the dissimilarity between *M. hispida* mucus and the corresponding seawater were then evaluated by SIMPER analysis (Table 1). Some microorganisms, such as members of the Pseudoalteromonadaceae, were the major OTUs that significantly constituted the general profiles observed for both bacteriomes, mucus and seawater (21.86%). However, other bacteria were specifically correlated with the mucus bacteriome, including members of the Desulfovibrionaceae (6%) and Flavobacteriaceae (3.6%), *Pseudoalteromonas* (2.76%), *Arcobacter* (2.64%), *Rugeria* (3.28%), and other genera of Rhodobacteraceae (2.15%).

**Table 1:** SIMPER analysis results, showing top 23 operational taxonomic units (OTUs) responsible for 83.55% dissimilarity between *Mussismilia hispida* mucus and seawater

OTUs	Mucus	Seawater	OTU contribution (%)^b^	Cumulative contribution (%)
	Average abundance^a^	Average abundance^a^		
Unclassified Pseudoalteromonadaceae	2,203.67	10,112.13	21.86	21.86
Unclassified Desulfovibrionaceae	2,253.75	158.88	6.01	27.87
Unclassified Flavobacteriaceae	1,396.42	8.13	3.64	31.51
*Alteromonas*	45.83	1,417.5	3.58	35.1
***Ruegeria*** *	**1,189.92**	372.75	3.28	38.38
*Vibrio*	276.67	1,247.75	2.83	41.21
*Pseudoalteromonas*	1,090.67	100.63	2.76	43.97
*Arcobacter*	1,010.17	2.38	2.64	46.61
Unclassified Desulfobulbaceae	847.92	1.25	2.22	48.82
Unclassified Desulfovibrionaceae	825.17	41.25	2.19	51.02
*Idiomarina*	76.58	817.13	2.17	53.18
**Unclassified Rhodobacteraceae**	**910.33**	276.5	2.15	55.34
Unclassified Hyphomonadaceae	804.17	2.75	2.1	57.44
*Halomonas*	560.92	122.13	1.64	59.08
*Thalassospira*	563.33	69.25	1.56	60.64
*Alcanivorax*	18.42	600.38	1.53	62.16
*Marinobacter*	10.92	543.63	1.41	63.57
Unclassified Vibrionaceae	613.17	153.75	1.28	64.85
Unclassified Acidaminobacteraceae	476.67	0	1.25	66.09
Unclassified Flavobacteriaceae	398.25	15.75	1.03	67.13
*Oceanicaulis*	317	141.38	0.97	68.1
*Idiomarina*	35.08	389	0.92	69.02
*Marinobacter*	0.17	334.25	0.87	69.9
***Phylum***				
Proteobacteria			63.97	63.97
Firmicutes			1.25	65.22
Bacteriodetes			4.67	69.89

a Mean abundance of each OTU.

b Contribution of each taxon to the overall dissimilarity between Mucus and seawater groups.

OTUS which are also observed in co‐occurrence analysis.

### 3.2 Bacterial 16S rRNA gene copy and MGE abundances

**Figure 2:**
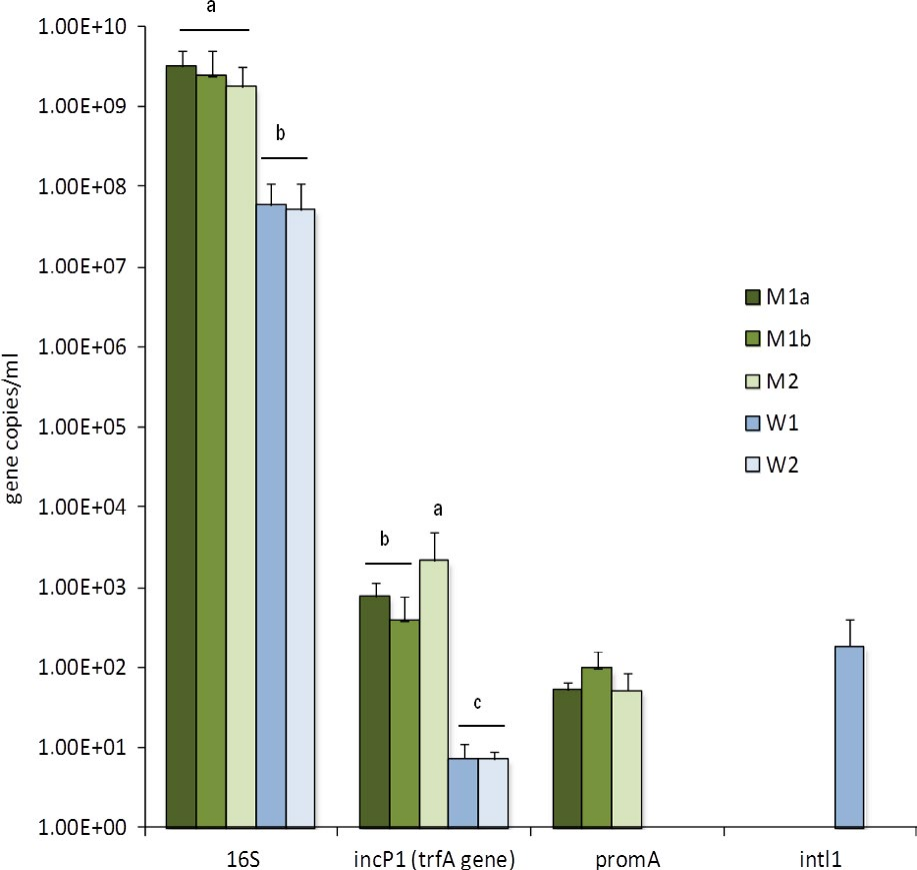
Abundances of 16S rRNA, *trfA*,* promA* and *intl1* genes from *Mussismilia hispida* mucus and surrounding seawater at Recife Itassepocu (site 1) and Recife de Fora (site 2). Column labels are as follows: M2: *M. hispida* mucus at site 2, M1b: *M. hispida* mucus at site 1—colonies without white spots, M1a: *M. hispida* mucus at site 1—colonies with white spots, W1: seawater at site 1, W2: seawater at site 2

In the coral mucus samples compared to the seawater samples, a higher abundance was seen for 16S rRNA (1.7–3.1e+09 gene/ml for mucus and 5.1–5.8e+07 gene/ml for seawater; *p *<* *.01), IncP‐1β (3.9e+02–2.1e+03 gene/ml for mucus and 7.1–7.4e+00 gene/ml for seawater; *p *<* *.01), and PromA (5.1–9.7e+01 gene/ml for mucus; *p *<* *.01) plasmid groups. This represents an increase in gene copies in mucus samples of 21% for 16S rRNA genes and 252% and 100% for plasmid groups (IncP‐1 and PromA, respectively), compared to the seawater samples. PromA plasmid groups were detected only in the coral mucus and were below the detection level in the seawater bacteriomes. No significant differences (*p* > .05) were observed between healthy colonies and those with morphological alterations for the 16S rRNA and plasmid groups (IncP‐1 and PromA). Copies of the *intl1* gene (Integron class I) were detected only in seawater, specifically from site 1, closer to the river mouth, while incW plasmid groups were not detected in any of the samples (Figure 2).

### 3.3 Coral core bacteriome and bacterial co‐occurrence in mucus

The core bacteriome of the *M. hispida* mucus (OTUs shared among all samples) was composed mostly of Proteobacteria (between 90% and 98%), followed by Firmicutes (Bacillaceae) (Table 2, Figure S2). Some of the OTUs that constituted the mucus core of *M. hispida* were also observed in the co‐occurrence analyses (Table 2, Figure S4), such as *Pseudomonas*,* Bacillus*,* Pseudoalteromonas*, Pseudoalteromonadaceae, and Rhodobacteraceae.

**Table 2:** Operational taxonomic unit (OTU) table of the bacterial core community associated with *Mussismilia hispida* mucus at Recife Itassepocu (site 1) and Recife de Fora (site 2)

OTU ID	Samples												ID used in this paper	Taxonomy				
	M1a (i)	M1a (ii)	M1a (iii)	M1a (iv)	M1b (i)	M1b (ii)	M1b (iii)	M1b (iv)	M2 (i)	M2 (ii)	M2 (iii)	M2 (iv)		Phylum	Class	Order	Family	Genera
540269	52	6,861	41	5	4	3	34	4	7	8	4	37	Exiguobacteraceae_1[Link]	Firmicutes	Bacilli	Bacillales	Exiguobacteraceae	
NR_OTU64	36	114	6	1	4	1	37	42	1	3	1	25	Bacillaceae[Link]	Firmicutes	Bacilli	Bacillales	Bacillaceae	
1078248	558	423	145	20	41	33	412	123	47	43	3	177	Bacillus^a^	Firmicutes	Bacilli	Bacillales	Bacillaceae	Bacillus
1087298	24	7	13	10	13	41	2	6	12	13	6	21	Alphaproteobacteria	Proteobacteria	Alphaproteobacteria			
309877	2,517	7	1	2	2	2	2	5	55	3,316	3,017	12,340	Hyphomonadaceae_1	Proteobacteria	Alphaproteobacteria	Rhodobacterales	Hyphomonadaceae	
829814	9,478	278	3,268	2,561	104	5,062	12	16	461	1,316	1,223	716	Rhodobacteraceae_5	Proteobacteria	Alphaproteobacteria	Rhodobacterales	Rhodobacteraceae	
1101488	2,378	15,366	131	491	12	9,864	2	1	172	340	309	305	Ruegeria	Proteobacteria	Alphaproteobacteria	Rhodobacterales	Rhodobacteraceae	Ruegeria
2932342	11	316	782	45	19,637	12,563	66	44,095	3	2	1	57	Desulfovibrionaceae_3[Link]	Proteobacteria	Deltaproteobacteria	Desulfovibrionales	Desulfovibrionaceae	
51975	5	7	1	1	3	47	9	4	18,923	2,193	1,837	31	Arcobacter[Link]	Proteobacteria	Epsilonproteobacteria	Campylobacterales	Campylobacteraceae	Arcobacter
823476	258	146	10	35	8	102	193	65	56	16	23	220	Alteromonas[Link]	Proteobacteria	Gammaproteobacteria	Alteromonadales	Alteromonadaceae	Alteromonas
812024	16	1	18	14	23	248	34	14	15	15	5	13	Glaciecola_2	Proteobacteria	Gammaproteobacteria	Alteromonadales	Alteromonadaceae	Glaciecola
509913	1,249	3	143	45	24	26	6	4	8	29	17	7	Marinobacter_1[Link]	Proteobacteria	Gammaproteobacteria	Alteromonadales	Alteromonadaceae	Marinobacter
956811	5	15	126	135	1,314	28	1	6	375	33	21	85	Idiomarina_3[Link]	Proteobacteria	Gammaproteobacteria	Alteromonadales	Idiomarinaceae	Idiomarina
141607	33	14	28	87	3	4	33	12	7	340	237	38	Idiomarina_1[Link]	Proteobacteria	Gammaproteobacteria	Alteromonadales	Idiomarinaceae	Idiomarina
182418	101	61	25	1	5	15	99	26	26	1	4	85	Pseudomonas^a^	Proteobacteria	Gammaproteobacteria	Pseudomonadales	Pseudomonadaceae	Pseudomonas
820978	5,005	8,329	3,379	341	3,846	1,047	15,048	2,042	1,445	183	341	5,630	Pseudoalteromonadaceae_6[Link]	Proteobacteria	Gammaproteobacteria	Vibrionales	Pseudoalteromonadaceae	
785565	386	519	238	23	312	78	443	154	100	14	37	367	Pseudoalteromonadaceae_4[Link]	Proteobacteria	Gammaproteobacteria	Vibrionales	Pseudoalteromonadaceae	
160928	144	162	136	19	126	31	422	48	18	3	5	142	Pseudoalteromonadaceae_1[Link]	Proteobacteria	Gammaproteobacteria	Vibrionales	Pseudoalteromonadaceae	
217623	15	22	19	2	70	19	78	225	24	1	2	21	Pseudoalteromonadaceae_3[Link]	Proteobacteria	Gammaproteobacteria	Vibrionales	Pseudoalteromonadaceae	
NCUR_OTU23335	68	19	3	1	13	6	1	1	9	40	44	194	Pseudoalteromonadaceae_7[Link]	Proteobacteria	Gammaproteobacteria	Vibrionales	Pseudoalteromonadaceae	
198609	27	38	19	2	34	10	130	34	30	1	4	35	Pseudoalteromonadaceae_2[Link]	Proteobacteria	Gammaproteobacteria	Vibrionales	Pseudoalteromonadaceae	
NR_OTU80	27	12	21	5	23	7	65	13	7	1	3	20	Pseudoalteromonadaceae_8[Link]	Proteobacteria	Gammaproteobacteria	Vibrionales	Pseudoalteromonadaceae	
821550	36	67	329	184	533	93	273	34	5	17	24	7	Pseudoalteromonas_6[Link]	Proteobacteria	Gammaproteobacteria	Vibrionales	Pseudoalteromonadaceae	Pseudoalteromonas
830290	646	483	6,481	8,569	10,253	1,140	980	143	1	167	151	24	Pseudoalteromonas_7[Link]	Proteobacteria	Gammaproteobacteria	Vibrionales	Pseudoalteromonadaceae	Pseudoalteromonas
939811	300	145	664	254	2,151	2,987	235	735	398	2,814	2,340	3,161	Vibrionaceae_4[Link]	Proteobacteria	Gammaproteobacteria	Vibrionales	Vibrionaceae	
837366	25	10	52	30	283	302	21	32	21	366	272	299	Vibrionaceae_3[Link]	Proteobacteria	Gammaproteobacteria	Vibrionales	Vibrionaceae	
NCUR_OTU15773	1	3	27	21	54	15	22	10	4	5	1	1	Vibrio_3	Proteobacteria	Gammaproteobacteria	Vibrionales	Vibrionaceae	Vibrio
792393	737	573	201	86	65	209	2,213	361	211	56	67	695	Vibrio_2[Link]	Proteobacteria	Gammaproteobacteria	Vibrionales	Vibrionaceae	Vibrio

M2, Mussismilia hispida mucus at site 2; M1b, Mussismilia hispida mucus at site 1—colonies without white spots; M1a, Mussismilia hispida mucus at site 1—colonies with white spots.

a OTUS which are also observed in co‐occurrence analysis.

Members of the bacterial core that are vertically transmitted in M. hispida, as described by Leite et al., 2017.

**Figure 3:**
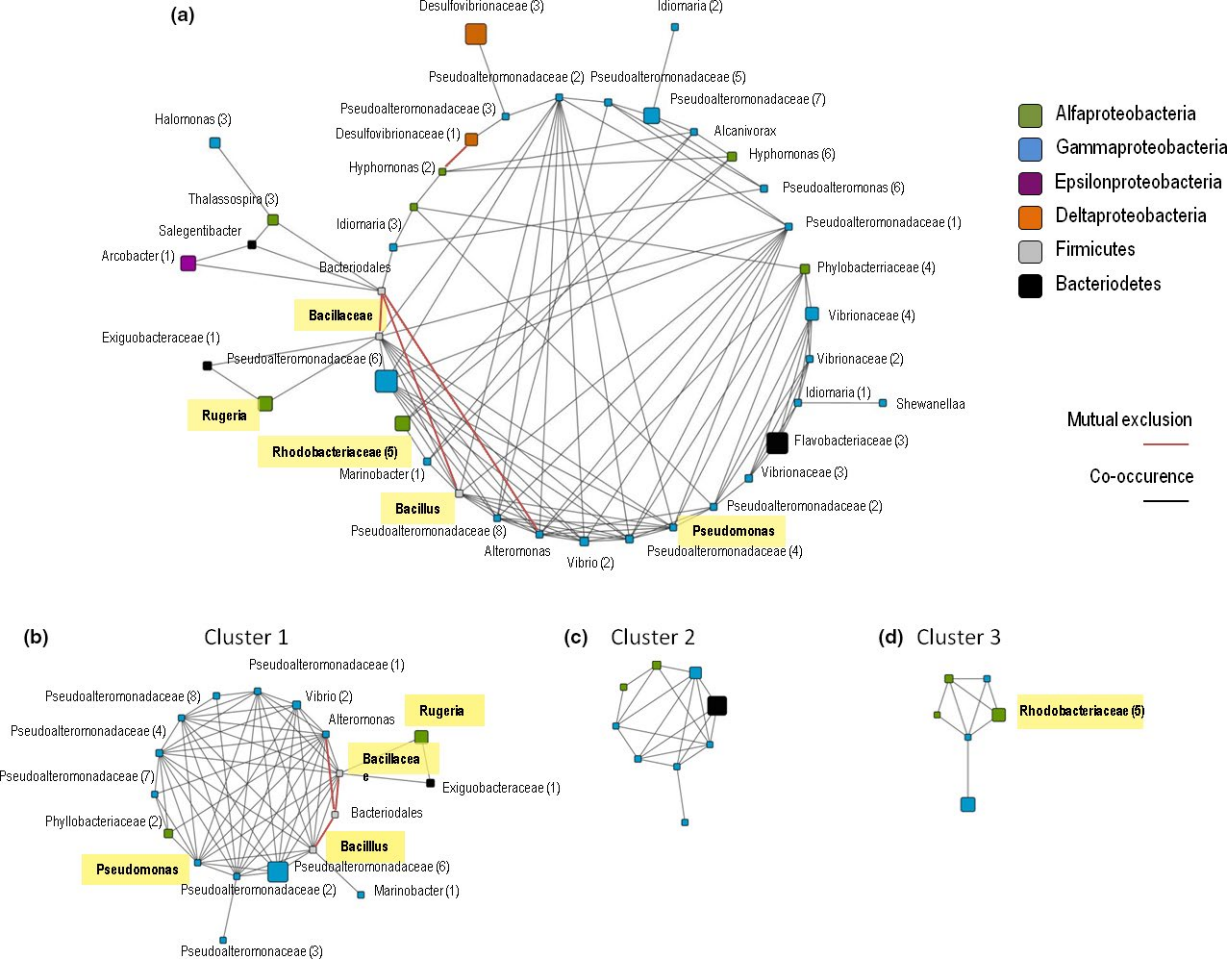
Network analysis of interactions among the more abundant bacterial operational taxonomic units (OTUs). Significant interactions among bacterial genera in *Mussismilia hispida* mucus from Recife Itassepocu (Site 1) and Recife de Fora (Site 2). Red lines indicate negative interactions (mutual exclusions), and black lines indicate positive interactions (co‐occurrences). The size of the nodes reflects the relative abundance of the genus in the entire data set, and the nodes are sorted and colored by phylum. (a) All significant interactions, involving 38 OTUs, sorted by class and densely interconnected regions, Cluster 1 (b), Cluster 2 (c), and Cluster 3 (d). The core bacteriome members vertically transferred (Leite et al., 2017) are highlighted in yellow

**Figure 4:**
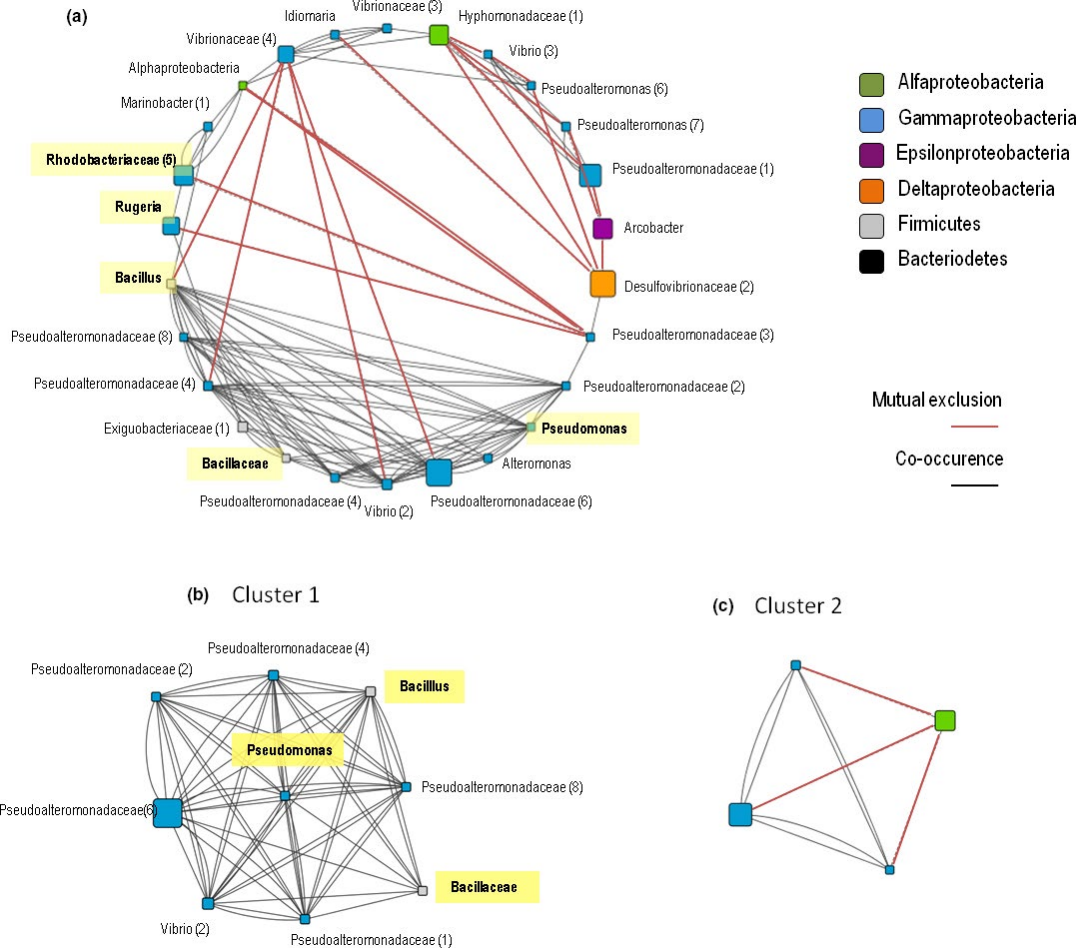
Network analysis of interactions in the core bacteriome. Significant interactions between bacterial genera in *Mussismilia hispida* mucus from Recife Itassepocu (Site 1) and Recife de Fora (Site 2). Red lines indicate negative interactions (mutual exclusions), and black lines indicate positive interactions (co‐occurrences). The size of the nodes reflects the relative abundance of the genus in the entire data set, and the nodes are sorted and colored by phylum. (a) All 121 significant interactions, involving 26 OTUs, sorted by class and densely interconnected regions, Cluster 1 (b) and Cluster 2 (c). The core bacteriome members vertically transferred (Leite et al., 2017) are highlighted in yellow

The co‐occurrence patterns of bacterial genera revealed that for the most abundant OTUs, a total of 139 significant co‐occurrences (*p *=* *.05) were detected between 38 bacterial OTUs in a matched subset of mucus samples from sites 1 and 2 (Figure 3a). For the bacterial core, a total of 121 significant co‐occurrences (*p *=* *.05) were detected between 26 bacterial genera (Figure 4a). For both, most abundant OTUs and bacterial core, most of the co‐occurrences were positive among the OTUs, and most of the correlated members belonged to the Proteobacteria, with some Firmicutes (Bacillaceae) also found. Cluster 1, consisting of the most abundant OTUs and the core bacteriome, included core members, such as members of OTUs from the genera *Pseudomonas* and *Bacillus* (Figures 3b and 4b). The other clusters (Figures 3c,d and 4c) were composed mostly of Proteobacteria.

The co‐occurrence of taxa, considering the most dominant OTUs in the total mucus (Figure 3) and the bacterial core OTUs (Figure 4), consisted mainly of the families Bacillaceae and Pseudoalteromonadaceae and the genera *Pseudomonas, Bacillus, Alteromonas,* and *Vibrio*. Most of the inter‐relationships within the bacteriome core OTUs were also related to the same groups, except for *Alteromonas* (Figures 3 and 4, Table 3). OTUs related to *Pseudomonas* and Pseudomonadaceae, and *Bacillus* and Bacillaceae were the most important groups showing positive correlations (Table 3).

**Table 3:** Top 10 interactions among the operational taxonomic units (OTUs) from the network analysis (Cluster 1) of the (A) most abundant OTUs and (B) core bacteriome of mucus at Recife Itassepocu (site 1) and Recife de Fora (site 2)

OTUs	More abundant OTUs	
	Number of positives correlations	Number of negative correlations
(A)		
Bacillaceae	12	1
*Pseudomonas*	11	—
**Pseudoalteromonadaceae**	11	—
**Pseudoalteromonadaceae**	11	—
***Alteromonas***	11	—
**Pseudoalteromonadaceae**	10	—
**Pseudoalteromonadaceae**	10	—
*Bacillus*	**10**	**1**
***Vibrio***	9	—
**Pseudoalteromonadaceae**	9	—
(B)		
*Pseudomonas*	16	—
**Pseudoalteromonadaceae**	16	—
**Pseudoalteromonadaceae**	15	—
*Bacillus*	15	—
**Pseudoalteromonadaceae**	15	—
***Vibrio***	15	—
**Pseudoalteromonadaceae**	14	—
**Pseudoalteromonadaceae**	13	—
Bacillaceae	8	—

**Figure 5:**
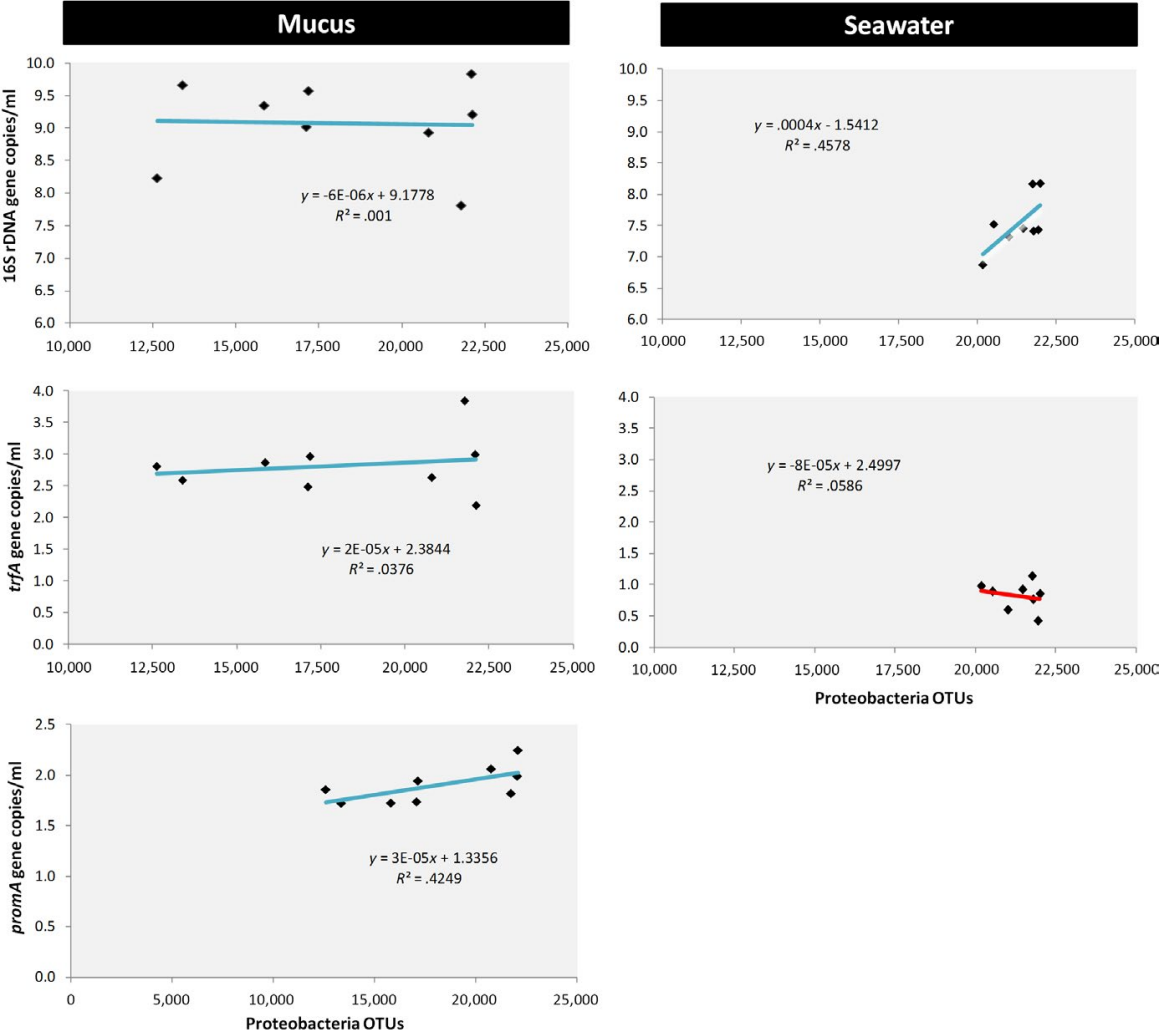
Correlation plot showing the functional correlation between Proteobacteria operational taxonomic units (OTUs) and 16S rRNA, *trfA*, and *promA* genes/ml for coral mucus and seawater bacteriome

The functional correlation between the abundances of Proteobacteria (OTUs) and 16S rRNA, *trfA,* and *promA* (genes/ml) in coral mucus and seawater bacteriomes generated different patterns. For mucus, no correlation (Pearson's *r *=* *.06) could be observed for the abundance of 16S rRNA copies and Proteobacteria OTUs. However, a positive correlation (Pearson's *r *=* *.54) was observed for seawater. Regarding the correlation between all tested plasmids and Proteobacteria OTUs, a positive correlation was observed (Pearson's *r:* trfA = 0.36 and promA = 0.62) for mucus samples, while a negative correlation was found for seawater samples (Pearson's *r *= −.15) (Figure 5).

## 4 DISCUSSION

Here, we describe the diversity and intercorrelations of the bacterial diversity, as well as the prevalence of MGEs associated with mucus from *M. hispida* and the surrounding seawater. We focused particularly on the BHR plasmid groups IncP‐1 and PromA, widely used as BHR plasmid proxies and indicated as the major providers of bacterial HGT in some soil environments (van der Auwera et al., 2009; Heuer & Smalla, 2012; Zhang et al., 2014), as well as on integron1, a good proxy for pollution (Gillings et al., 2015). The use of these plasmid groups was also based on their hypothesized importance, which is related to resistance to antibiotics and heavy metals and to the efficient mobilization among Gram‐negative bacteria.

Our main findings indicate that key groups of bacteria, that is, Proteobacteria, followed by Firmicutes, mainly represented by members of Rhodobacteraceae and the genera *Pseudomonas* and *Bacillus* associated with *M. hispida*, were present in the coral mucus. These dominant groups and members of the entire *M. hispida* microbial core have also been described as being vertically transmitted from parent to offspring in the same coral species (Leite et al., 2017). In addition, this core microbiome and some other groups in the mucus bacteriome have a positive relationship of co‐occurrence, especially the families Desulfovibrionaceae and Flavobacteriaceae, the genera *Pseudoalteromonas* and *Arcobacter*, and *Rugeria* and other members of Rhodobacteraceae, suggesting that these microorganisms could be selected by the holobiont. Our data also indicated that the holobiont selects (i.e., contains a higher abundance or even a specific persistence) the IncP‐1 and PromA groups of BHR plasmids, as these were significantly more abundant in coral mucus or absent in the seawater samples, respectively.

Studies on coral microbiomes have shown the importance of these organisms for host health, fitness, maintenance (Musovic, Oregaard, Kroer, & Sørensen, 2006; Peixoto et al., 2017; Santos et al., 2014, 2015; Sweet & Bulling, 2017; Webster & Reusch, 2017), and evolution (Bhattacharya et al., 2016). Microbial surveys have contributed to our understanding of how microbial communities can promote the resilience of coral reefs to environmental stress (Bhattacharya et al., 2016; Peixoto et al., 2017; Sweet & Bulling, 2017; Webster & Reusch, 2017), and have generated knowledge of the beneficial potential of the microbiome and its potential future manipulations (Damjanovic et al., 2017; Peixoto et al., 2017; Sweet & Bulling, 2017; Webster & Reusch, 2017), thereby improving the health of reef ecosystems. Knowledge of key coral microbiome microbial groups and potential intertaxa correlation patterns can improve and guide such BMC manipulations, by indicating stable, well‐adapted populations that could be involved in beneficial mechanisms and would be, at the same time, competitive, and well established in manipulative approaches.

The seawater bacteriome observed here was more diverse than the coral bacteriome, as reported in other studies (Castro et al., 2010; Garcia et al., 2013; Reis et al., 2009; Rojo, 2010; Rosenberg, Kellogg, & Rohwer, 2007). *Mussismilia hispida* bacterial communities from the mucus samples were composed mainly of Proteobacteria, a phylum that is widely found in *M. hispida* microbiomes (Castro et al., 2010; Leite et al., 2017; Lins‐De‐barros et al., 2010; Musovic et al., 2006) as well as in other species of the genus *Mussismilia* (Fernando et al., 2015; Garcia et al., 2013; Santos et al., 2015) and in other coral genera (Bourne & Munn, 2005; Kimes, van Nostrand, Weil, Zhou, & Morris, 2010; Kimes et al., 2013; Mouchka et al., 2010; Vega Thurber et al., 2009). Moreover, the phylum Proteobacteria is quite abundant in a range of *Mussimilia* microhabitats such as the mucus, tissue, and skeleton, compared with other bacterial groups (Castro et al., 2010; Fernando et al., 2015; Garcia et al., 2013; Leite et al., 2017; Lins‐De‐barros et al., 2010; Reis et al., 2009; Santos et al., 2015).

Recent studies have suggested that the coral microbial community is composed of both a stable and a variable fraction. The stable fraction is proposed to be directly involved in basic host requirements (i.e., the microbial core, which is also composed of two components, a host‐specific ubiquitous community, and a niche‐specific community). The variable fraction is proposed to vary rapidly with environmental shifts (Hernandez‐Agreda, Leggat, Bongaerts, & Ainsworth, 2016). For the maintenance of the coral holobiont, the host can acquire its symbionts directly, via parental gametes/eggs (i.e., vertical transmission (Musovic et al., 2006; Sharp, Distel, & Paul, 2011; Padilla‐Gamiño et al., 2012)) or through acquisition from the surrounding environment (i.e., horizontal transmission) (Apprill, Marlow, Martindale, & Rappé, 2009; Knowlton & Rohwer, 2003). The early acquisition and maintenance of a microbiome may ensure the establishment of key mechanisms to protect and foster the settlement and development of coral larvae (Lema et al., 2014; Sharp & Ritchie, 2012).

Leite et al. (2017) have indicated that members of the core bacteriome of *M. hispida* (i.e., the genera *Burkholderia, Pseudomonas, Acinetobacter, Ralstonia, Inquilinus* and *Bacillus,* and unclassified Rhodobacteraceae) were transmitted vertically to offspring, through the gametes, reinforcing the potential importance of the coral bacteriome core members. Therefore, we have focused on the core bacteriome from the *M. hispida* mucus samples from different sampling points. Our data have also indicated core members that have been described by Leite et al. (2017) at early life stages, such as *Pseudomonas, Bacillus,* and Rhodobacteraceae members, in all coral mucus samples from the two sampling sites, and showing a high level of intertaxa relationships. In addition, considering the BHR that were screened, the IncP‐1 plasmid group was the most abundant plasmid group in the coral mucus bacteriome. These plasmids have a wide distribution and are highly efficient for Gram‐negative bacteria (Popowska & Krawczyk‐Balska, 2013), but have also been reported mobilizing Gram‐positive bacteria (Musovic et al., 2006). This group of plasmids can exchange a wide range of potentially advantageous genes, such as genes for antibiotic resistance and degradation of different carbon sources (Popowska & Krawczyk‐Balska, 2013; Shintani et al., 2010; Zhang et al., 2014), which, given the abundance of “enriched” plasmids, could suggest a key role of HGT in the coral–microbiome interactions.

The second most abundant group of plasmids, PromA, proposed by van der Auwera et al. (2009), was detected only in coral samples. Previous studies have found that IncP‐1 and PromA, BHR groups of plasmids, are extremely important gene carriers in other systems, such as for soil bacterial communities (van der Auwera et al., 2009; Heuer & Smalla, 2012; Zhang et al., 2014), and are both efficient plasmids for gene exchanges between members of the Proteobacteria group (Zhang et al., 2014). We find it interesting that this group was detected only in coral mucus samples. Although there are multiple possible explanations, this could also indicate that the holobiont can indeed select and concentrate a specific diversity of MGEs.

When considering the network analyses from the total mucus samples, that is, not considering only the bacteriome core, we have found a large number of related OTUs, mainly based on co‐occurrence among Proteobacteria and Firmicutes members. More specifically, separate clusters harboring core microbiome members, previously described as vertically transmitted in *M. hispida* (*Pseudomonas*,* Bacillus* and Rhodobacteraceae members) (Leite et al., 2017), were observed. There are multiple possible explanations for these patterns of co‐occurrence and dominance, and we discuss a few of them below. One possibility is that taxonomic relationships, at the OTU level, are indeed relevant for the coral microbiome assembly. In this case, it could suggest that something about these specific taxa (e.g., key functions), that is best, or exclusively, provided by these members, could not be replaced by other taxa or HGT. This could explain the stable taxonomic selection observed. Alternatively, or complementarily, interactions between taxa, or between the host and these taxa, maintain their presence or absence and the observed correlations. In addition, these patterns could also be merely a consequence of history, that is, successive vertical transmission of specific groups that leads to correlations.

The observed co‐occurrence and specific taxonomic persistence indicate that these taxa are potential key players in coral health, give that their presence in the offspring is ensured. This co‐occurrence and taxonomic persistence could also suggest that these members might be actively involved in the persistence of other bacterial groups through symbiotic relationships. On the other hand, these data could indicate that these coexisting and dominant groups are independently influenced by environmental factors. Thus, these groups would be selected as the most able to survive in this environment (Barberán et al., 2012), due to their potential key irreplaceable functions. In this case, they are only sharing the *M. hispida* mucus niche. Nevertheless, this would mean that these are the most competitive groups within this niche, which clearly indicates them as important targets for *M. hispida* BMC manipulative studies.

In parallel, positive correlations were observed between the coral mucus and the abundance and/or the specific presence of the screened plasmids. Although plasmid abundance is not supported by the observed stable bacterial taxonomic diversity, as, in this case, the relevant role seems to be related to the taxonomic level rather than to transferrable functions, it could be related to the variable fraction of the coral microbiome. Thus, it is possible that these “holobiont‐enriched” plasmids could be in fact associated with the noncore, non‐co‐occurring taxa. The “enriched” presence of these MGEs within the holobiont could indicate that advantageous genes could be eventually exchanged between all members of the coral bacteriome. This advantageous exchange of genes could eventually support the transient (and even the stable) members under adverse conditions, which can, in turn, contribute to the resilience of the host in the face of environmental shifts. However, Hall, Williams, Paterson, Harrison, and Brockhurst (2017) have recently suggested that conjugation can be reduced by positive selection, indicating that HGT can be inhibited by those beneficial elements. The conjugative mobilization would be more related to infections and parasitic elements. Thus, the remaining questions are as follows: To what taxa do these “enriched” plasmids belong? And is there active HGT, mediated by these plasmids, occurring?

Taken together, the high prevalence of co‐occurrence between core bacterial groups and the specific plasmid‐pattern data could suggest separate roles in the coral bacterial assembly. It is also possible that both mechanisms could be correlated, as a random consequence of the high prevalence of the dominant microbial diversity, Proteobacteria. This could, in turn, randomly select those plasmids that can be established by the abundance of this dominant group, though not being necessarily relevant for this dominance. This suggestion is supported by the correlations between Proteobacteria OTUs and plasmids in the mucus samples. On the other hand, this role could be associated with specific mechanisms, evolved to selectively permit the persistence of the dominant components of the bacteriome and associated plasmids, which could allow eventual cooperation between other (and transient) members, mediated by gene exchange. Both hypotheses are somehow driven by the holobiont and its microbial diversity.

## CONFLICT OF INTEREST

The authors declare no conflict of interest.

## AUTHORS’ CONTRIBUTIONS

RSP, DCAL and ENC performed study conception and design. DCAL and ENC performed acquisition and identification of coral samples. DCAL, ENC performed acquisition of data (conducting of experiments). RSP, DCAL, JFS and JDE performed analyses and interpretation of data. RSP and DCAL drafted the manuscript. All authors critically revised the manuscript. RSP, JFS and JDE provided financial support.

## DATA ACCESSIBILITY

The raw data from each sample are available at the NCBI Sequence Read Archive (SRA) under Accession Numbers SRR5903387—SRR5903406.
