# Supplementary figures and images for: Specific plasmid patterns and high rates of bacterial co‐occurrence within the coral holobiont
Source: Ecol Evol. 2018 Jan 11;8(3):1818–32. doi: 10.1002/ece3.3717 (PMC5792611; doi:10.1002/ece3.3717)

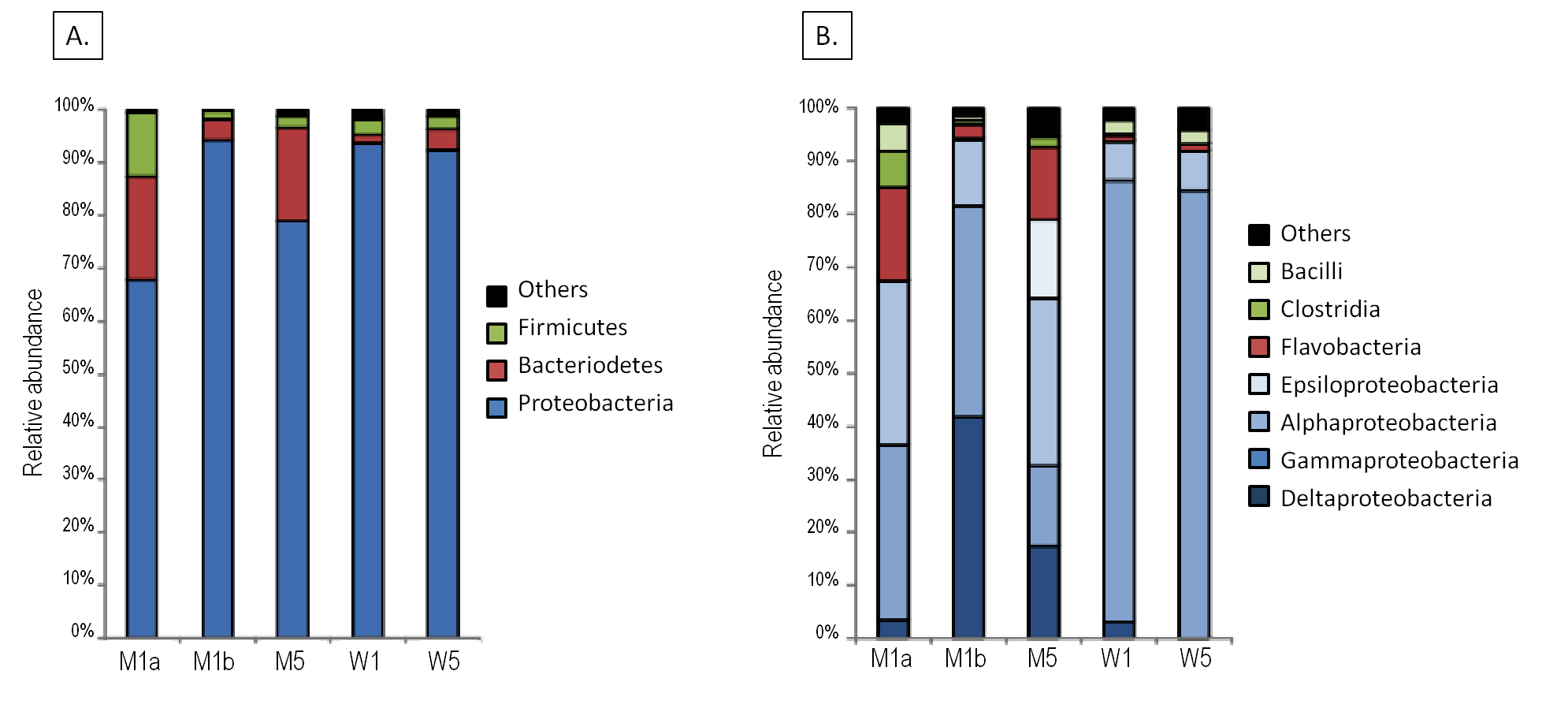

Supplement: Supplementary file 1 [file ECE3-8-1818-s001.tiff]

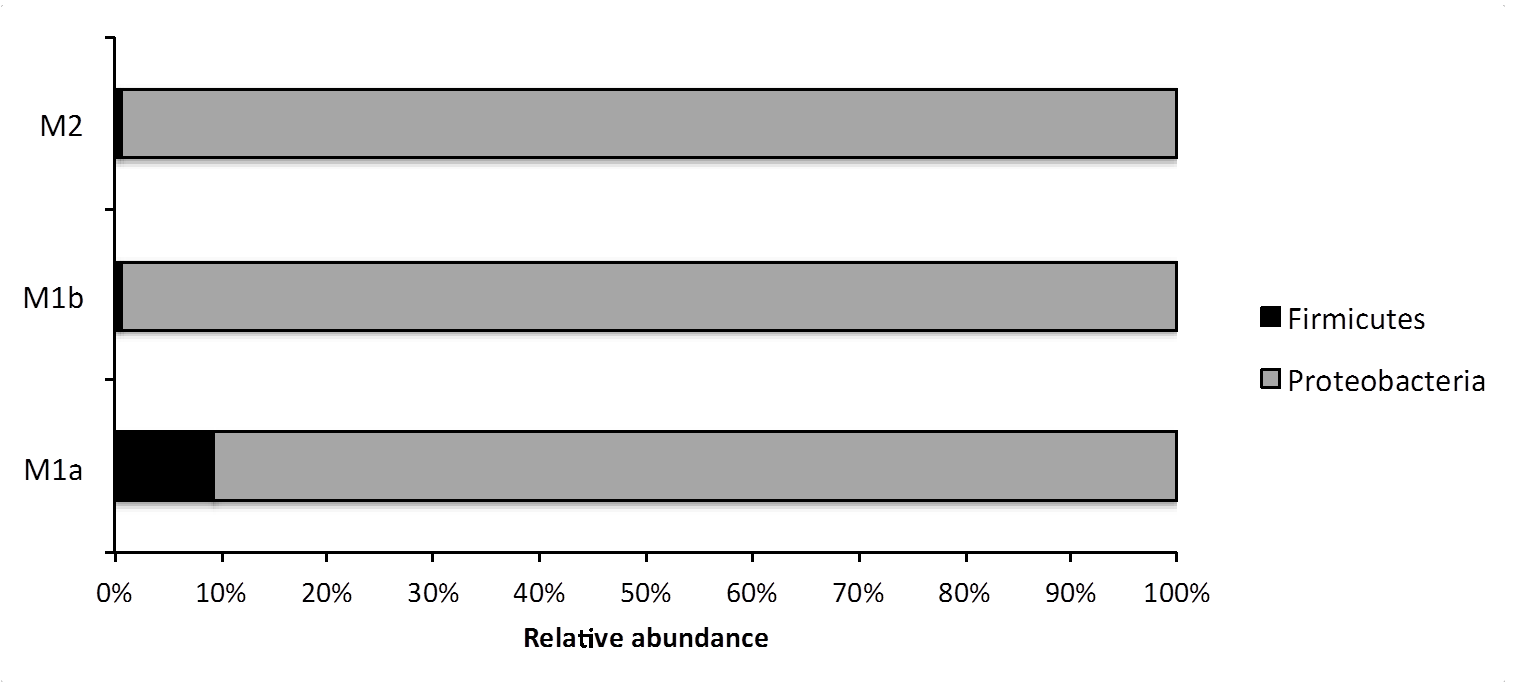

Supplement: Supplementary file 2 [file ECE3-8-1818-s002.tiff]

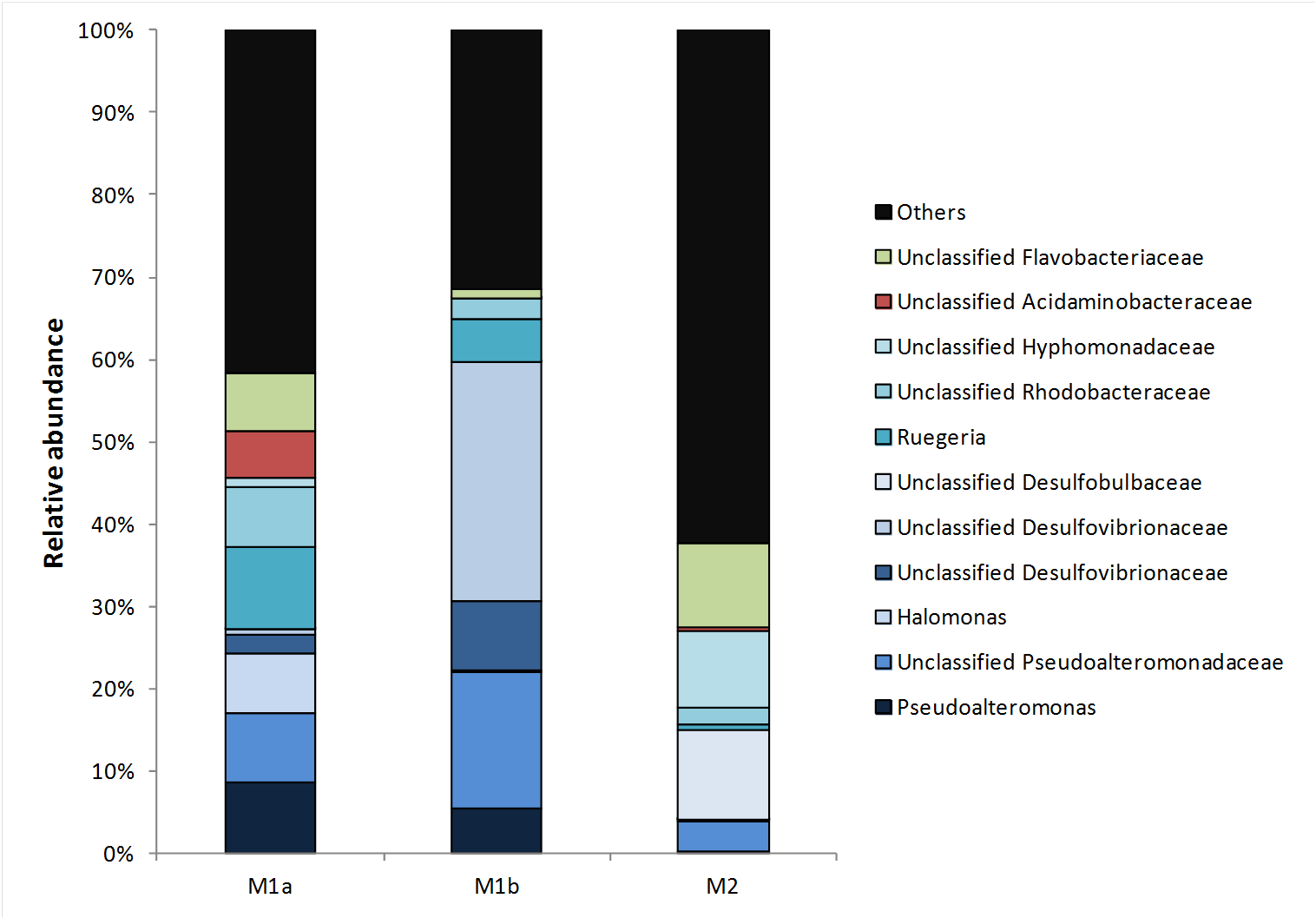

Supplement: Supplementary file 3 [file ECE3-8-1818-s003.tiff]

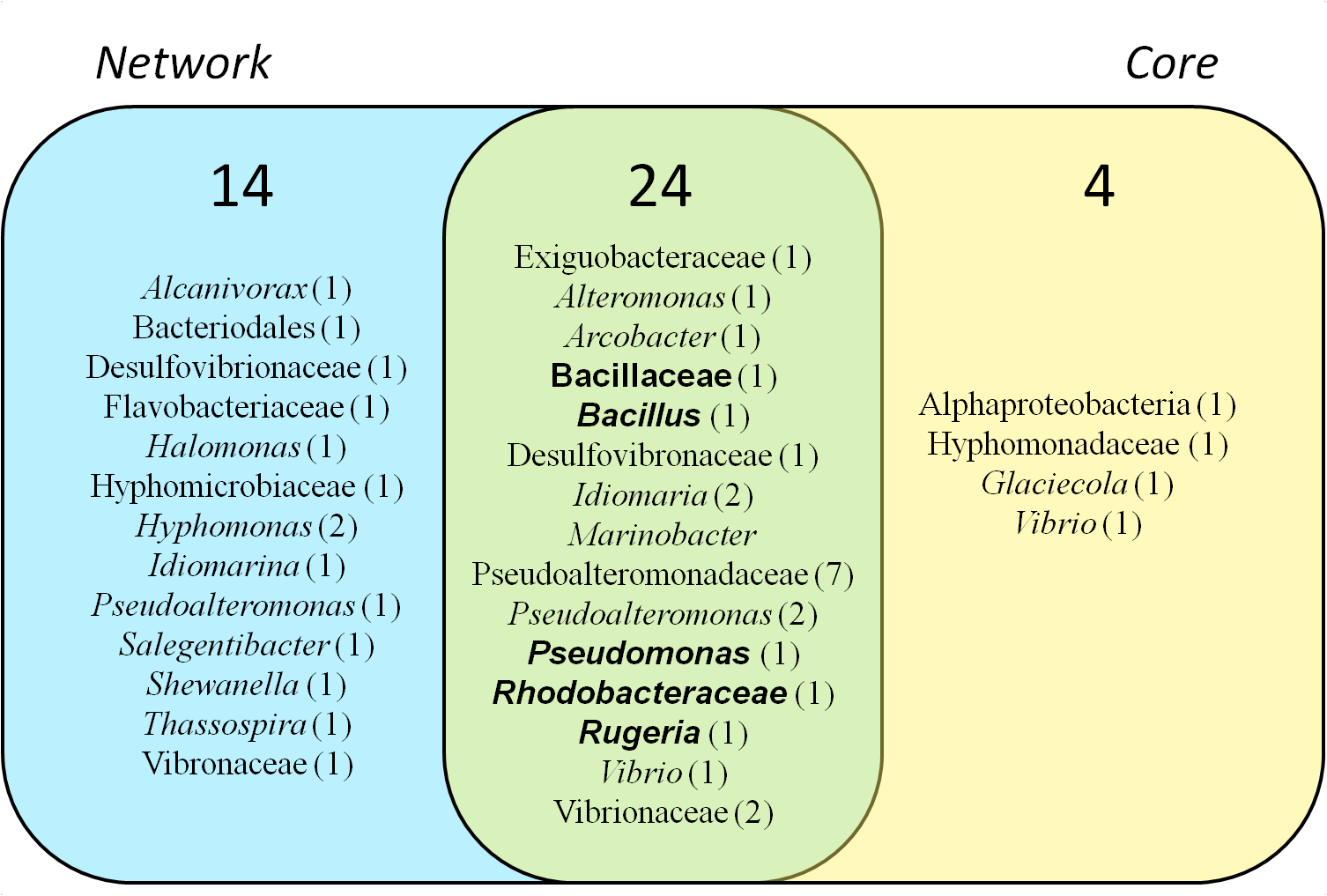

Supplement: Supplementary file 4 [file ECE3-8-1818-s004.tiff]
