# Supplementary material for: Specific plasmid patterns and high rates of bacterial co‐occurrence within the coral holobiont
Source: Ecol Evol. 2018 Jan 11;8(3):1818–32. doi: 10.1002/ece3.3717 (PMC5792611; doi:10.1002/ece3.3717)
Supplement: Supplementary file 5 [file ECE3-8-1818-s005.docx]

**Supplementary figures/tables**

**Table S1.**

| Target | Primers concentration (µM) | Annealing temp. (ºC) | Reference |
| --- | --- | --- | --- |
| Bacteria | 0.2 | 55 | Modified by Muyzer *et al.,* 1993 |
| IncP-1β group plasmids (*rep*A gene) | 0.8 | 59 | Zhang et al., 2015 |
| PromA group plasmids  (*trf*A2 gene) | 0.8 | 57 | Gotz et al., 1996 |
| IncW group plasmids  (*ori*V gene) | 0.5 | 59 | Gotz et al., 1996  Bahin et al 2008 |
| Integrons class I  (*intl*1 gene) | 0.5 | 60 | Stokes *et al.,* 2006 |
|  |  |  |  |

**Table S2**.

| Samples | Observed OTUs | PD | Chao |
| --- | --- | --- | --- |
| Mucus A – Site 1 | 274.61 ± 9.57 b | 13.63 ± 0.54 b | 226.33 ± 14.56 b |
| Mucus B – Site 1 | 177.98 ± 44.47 c | 9.90 ± 2.11 c | 138.60 ± 44.40 c |
| Mucus – Site 2 | 282.20 ± 33.17 b | 13.86 ± 1.08 b | 230.05 ± 24.05 b |
| Water – Site 1 | 347.23 ± 38.55 a | 18.55 ± 2.23 a | 308.60 ± 43.18 a |
| Water – Site 2 | 255.57 ± 12.17 b | 14.10 ± 0.30 b | 228.25 ± 7.23 b |
